# Occipital Neuralgia Treated With Acupuncture: A Case Report

**DOI:** 10.1177/2164956119890546

**Published:** 2019-11-20

**Authors:** Molly Mallory, Brent Bauer, Tony Chon

**Affiliations:** 1 Integrative Medicine and Health, Mayo Clinic, Rochester, Minnesota

**Keywords:** acupuncture, occipital neuralgia, integrative medicine, pain

## Abstract

We present a case report of a patient whose occipital neuralgia symptoms were substantially improved after a single acupuncture treatment with complete resolution after only a short course of care. The patient, a 78-year-old woman, presented to our institution’s neurology department with symptoms of posterior neck pain and electrical sensations in her head which had been present for more than 1 year. With a desire to avoid pharmacologic intervention or invasive procedures, the patient requested acupuncture treatment. The patient received 5 acupuncture treatments over the course of 8 days with substantial resolution of her head pain after a single treatment. This case is suggestive that acupuncture may be a beneficial treatment for patients with occipital neuralgia.

## Introduction

Occipital neuralgia is defined by the National Institute of Neurological Disorders and Stroke as a distinct type of headache characterized by piercing, throbbing, or electric-shock-like pain in the upper neck, back of the head, and behind the ears.^1^ Patients may experience pain in the scalp, forehead, and behind the eyes. Occipital neuralgia may be a product of pathology in the greater, lesser, or third occipital nerves.^2^ Treatments may include anti-inflammatory medications, preventive medication, such as tricyclic antidepressants, rest, massage therapy, and physical therapy. This case is suggestive that acupuncture may be an effective treatment strategy for occipital neuralgia and have a secondary benefit of improving insomnia, dizziness, and visual disturbance.

## Presenting Concerns

The patient was a 78-year-old woman with a medical history notable for chronic obstructive pulmonary disease, hyperlipidemia, diabetes mellitus type 2, obstructive sleep apnea, and headaches. She presented with episodic spells of posterior neck pain, brief electric shock sensations in the neck and head, visual disturbance, and dizziness. The spells were occurring 20 times per hour. She was unable to drive or read given the visual disturbance and blurred vision. Brain magnetic resonance imaging without contrast revealed a meningioma overlying the left frontal lobe with no acute findings. The meningioma had been present and stable for more than a decade. Brain and neck magnetic resonance angiogram revealed a 50% to 60% narrowing of the right common carotid artery and internal carotid artery, likely atherosclerotic in nature. She was evaluated by a neurologist who diagnosed occipital neuralgia. She was offered a medication trial (gabapentin) or occipital nerve blocks. She was not comfortable with either of these options and elected to try acupuncture instead.

## Clinical Findings

The patient presented for acupuncture treatment following her initial neurological consultation. She reported 1 year history of head and neck pain. She reported episodes of “zapping electrical shock” sensations that originate at the base of the skull and travel to the top of her head. She stated that the painful sensations are accompanied by a momentary visual disturbance, which she had difficulty describing but appeared to be a momentary blurring of vision. The visual disturbance prevented her from driving a car. Despite having multiple pair of prescription eyeglasses, she was unable to read. She reported that she could have 100 or more of these spells each day. She endorsed dizziness and sensations of lightheadedness.

## Therapeutic Focus and Assessment

### First Acupuncture Treatment (7/15/2019)

The patient presented for her initial acupuncture consultation and treatment in the outpatient clinical setting. She had stable vital signs, and she appeared alert and oriented. After providing informed consent, the patient rested in the supine position on the treatment table. Acupuncture points were selected based on Traditional Chinese Medical theory using both local and distal points on meridians that traverse the area of pain and dysfunction. Acupuncture points were chosen based on the Traditional Chinese Medical diagnosis of “Liver Yang Rising” and “Liver Blood Deficiency.”

The patient’s skin was cleaned with alcohol prior to needle insertion. Presterilized and single-use stainless steel acupuncture needles were inserted to acupoints: GB20, GB21, LI4, SJ5, GB34, SP6, LV5, and LV3. Needles were retained for 30 minutes. Upon completion of the acupuncture treatment, the patient reported feeling very relaxed with a reduction in the severity of her head pain.

### Second Acupuncture Treatment (7/18/2019)

The patient presented for her second acupuncture treatment stating that she “felt much better” immediately following her initial treatment. She reported a complete resolution of the occipital neuralgia including the electrical zapping sensations. She reported “a little bit” of neck discomfort. She denied visual disturbance with the exception of when lying on her right side. The original acupuncture treatment strategy and point selection were duplicated.

### Third Acupuncture Treatment (7/19/2019)

The patient presented for her third acupuncture treatment reporting a single and fleeting episode of visual disturbance the previous afternoon. She denied having any episodes of head pain. She reported an improvement with the quality of her sleep with fewer episodes of waking during the night. The original acupuncture treatment strategy and point selection were duplicated.

### Fourth Acupuncture Treatment (7/22/2019)

The patient presented for her fourth acupuncture treatment. She denied head or neck pain, dizziness, or a sense of lightheadedness. She reported “very few episodes” of visual disturbance. She reported that she was able to “spend some time reading again.” By her estimation, her symptoms had improved by 80%. The original acupuncture treatment strategy and point selection were duplicated.

### Fifth Acupuncture Treatment (7/23/2019)

The patient presented for her fifth acupuncture treatment. She reported a single “brief” episode of visual disturbance which occurred when she was lying down and rotated her head to the right. She denied head pain, neck pain, or dizziness. The original acupuncture treatment strategy and point selection were duplicated.

#### Follow-up and outcomes

The patient received 5 acupuncture treatments over the course of 8 days. Following the initial treatment, she reported a substantial resolution of her head pain, including the “zapping” sensations. She reported a significant resolution of her dizziness and blurry vision. Over the course of the next 7 days, she experienced only fleeting episodes of visual disturbance and head pain. She reported an improvement in the quality of her sleep. She was reportedly able to read the newspaper and magazines.

Following her third acupuncture treatment, the patient underwent magnetic resonance brain angiogram and magnetic resonance neck angiogram which demonstrated 40% narrowing of the mid right common carotid artery and a 50% to 60% diameter narrowing of the right internal carotid artery, likely atherosclerotic in nature. She then met with her Neurologist who suggested that the patient continue with acupuncture treatment given her substantial improvement since initiating treatment. The patient cancelled her scheduled cervical magnetic resonance imaging given that her symptoms had greatly improved.

## Discussion

The present case describes the clinical course of a patient presenting with symptoms suggestive of occipital neuralgia along with unexplained visual disturbance and dizziness. The patient found resolution of her symptoms following a short course acupuncture treatment with sustained resolution throughout the course of her treatment. The patient did not initiate or incorporate any new pain interventions during the course of acupuncture treatment.

Patients are increasingly seeking nonpharmacologic and noninvasive approaches to managing symptoms. Acupuncture has been shown to be a safe intervention when performed by trained professionals.^3^ Reported adverse events are rare and tend to be minor, such as bruising.^4^ Acupuncture is an effective treatment for pain. The pain-relieving effects of acupuncture may persist over time and cannot be interpreted as merely a placebo effect.^5^ In addition to treating pain, acupuncture has shown favorable results in a variety of conditions including nausea, anxiety, and the ability to cope with symptoms.^6^ A growing number of cancer patients are expressing interest in acupuncture to mitigate the side effects of conventional cancer treatment including nausea, fatigue, neuropathy, hot flashes, and mood disturbance.^7,8^ Oncology acupuncture, albeit somewhat of a new specialty, is showing promising results in managing cancer-related symptoms.^8^ Multiple symptoms can be addressed in a single acupuncture visit which is of benefit to the patient.

Acupuncture could prove to be a cost-saving method for the treatment of occipital neuralgia. The average cost of an acupuncture treatment ranges from $50 to $120. A course of treatment with acupuncture may consist of 4 to 8 treatments. Nerve blocks can easily cost far more, especially if repeated treatments are needed. Chronic prescriptions of medications, such as gabapentin, can easily cost as much or more as a full course of acupuncture with the added challenges of side effects for many patients.

Many traditions and combinations of styles of acupuncture and acupuncture practice exist. Precise treatment protocols are lacking with varied thoughts on the prescription for acupuncture.^9^ Current findings suggest that acupuncture dosing and frequency are factors to be considered when measuring efficacy.^10^ Future research in the form of a pragmatic trial comparing acupuncture to usual care for occipital neuralgia might also examine the optimal dosing of acupuncture including the timing, frequency and duration of treatment.
